# DOES MEMORY CARE MATTER? EXAMINING THE EFFECT OF DEMENTIA-LICENSED CARE ON RESIDENTS’ OUTCOMES

**DOI:** 10.1093/geroni/igz038.2008

**Published:** 2019-11-08

**Authors:** Portia Y Cornell, Momotazur Rahman, Wenhan Zhang, Kali Thomas

**Affiliations:** Brown University, Providence, Rhode Island, United States

## Abstract

The objective of this study is to estimate the effect of receiving care in a dementia-care licensed (DCL) assisted living community, versus a standard AL, on outcomes of residents with ADRD. In four states that issue a license for specialized dementia care (AL, CO, MS, and NY), we identify a cohort of 5,720 Medicare fee-for-services beneficiaries with ADRD who moved to an AL in 2014. To control for unobserved factors that contribute to a patient’s selection of AL type, we use the difference in the log-distances from an individual’s home address to the nearest DCL and standard AL as an instrumental variable. We will report the effect of residence in a DCL AL on mortality, inpatient hospital days, emergency department utilization, and hospice use, showing how the use the distance instrument offers differing estimates from unadjusted or multiple-regression methods.

